# Ovarian Dysfunction and Polycystic Ovary Syndrome in the U.S. Military Active Component, 2014–2023

**Published:** 2025-01-20

**Authors:** William Douthitt, Jessica H. Murray, Shauna L. Stahlman

**Affiliations:** 1 Uniformed Services University of the Health Sciences, Bethesda, MD; 2 Epidemiology and Analysis Branch, Armed Forces Health Surveillance Division, Defense Health Agency, Silver Spring, MD

## Abstract

This retrospective cohort study examined ovarian dysfunction diagnosis incidence among female active component service members in the U.S. military from 2014 to 2023 using medical encounter data from the Defense Medical Surveillance System. Ovarian dysfunction diagnosis incidence gradually increased during this period, driven almost entirely by polycystic ovary syndrome (PCOS), of which the incidence increased from 32.0 cases per 10,000 person-years in 2014 to 60.3 cases per 10,000 person-years in 2023. Increases occurred among all demographic subcategories. This study also assessed independent association between ovarian dysfunction and socio-demographic and medical covariates, including COVID-19 infection and vaccination status. History of obesity had the strongest association with PCOS incidence, with an adjusted incident rate ratio (aIRR) of 2.5 and 95% confidence interval (CI) of 2.3-2.6. COVID-19 infection was modestly associated with PCOS incidence (aIRR 1.2; 95% CI, 1.1-1.3). COVID-19 vaccination status was not independently associated with increased PCOS incidence. A potential contributing factor of increased PCOS diagnosis incidence is that recent updates to the diagnostic criteria enabled more clinical and telehealth diagnoses. The increased incidence may also reflect the increasing rate of obesity and other related health burdens in the U.S. military.

**What are the new findings?:**

Incidence of diagnosis of ovarian dysfunction, driven almost entirely by polycystic ovary syndrome, increased steadily among female active component service members from 2014 to 2023. This increase in incidence was observed in all demographic subgroups and had the strongest independent association with pre-existing obesity.

**What is the impact on readiness and force health protection?:**

Health conditions due to ovarian dysfunction cause significant morbidity for female service members and contribute to reduced readiness and increased military health care spending. Increases in polycystic ovary syndrome in recent years could manifest in negative career impacts, including disqualification from certain military occupations and fewer service women able to meet retention standards.

## 1 BACKGROUND

Ovarian dysfunction is not a specific condition, but instead refers to a diagnostic code that may cover a range of conditions in which ovaries fail to function properly, often leading to hormonal imbalances, reduced ovulation, and associated physiological complications. These conditions include excess estrogen and androgen levels, primary ovarian insufficiency, and polycystic ovary syndrome (PCOS), among others.

PCOS is one of the most common endocrine and metabolic disorders affecting women of reproductive age, with an estimated worldwide prevalence ranging from 8% to 20%.^1,2^ The 3 cardinal signs and symptoms of PCOS are 1) oligo-/amenorrhea, 2) hyperandrogenism, and 3) polycystic ovary morphology. The pathophysiology of PCOS is complex and remains incompletely understood, but environmental, genetic, and metabolic factors are assumed to be involved.^1,3,4^ Associations have been found between PCOS and reproductive hormone dysregulation, obesity, insulin resistance, high calorie diets, smoking, suboptimal exercise, and genetics.^1^ PCOS is a disqualifying condition for military recruitment,^5^ and its sequelae make it difficult to maintain readiness standards for retention.

Ovarian dysfunction conditions, in particular PCOS, can cause a range of issues for the U.S. military, as these conditions can be associated excess weight gain, menstrual dysregulation, decreased fertility, cognitive and mood disturbances, and immune and endocrine dysfunction.^1,3,6^ Excess weight gain can further place personnel at increased risk for musculoskeletal injuries, diabetes, heart disease, and sleep impairment.^7,8^ These outcomes can directly affect fitness test and body composition pass rates, deployability, and personnel recruitment and retention.^5,7,8^ Female active component service members (ACSMs) with PCOS have reported negative career impacts, particularly disqualification from career tracks such as aviation, submarines, diving, nuclear, and missile operation.^9^ There are still gaps, however, in understanding of PCOS’s full impacts on female ACSMs’ careers and health. A 2022 meta-analysis estimated the financial burden of PCOS to the overall U.S. health care system at $8 billion a year,^10^ which suggests that the condition may also present a significant cost burden to the Military Health System (MHS).

To further determine ovarian dysfunction trends in the U.S. military, this study’s primary objective was to describe the incidence of ovarian dysfunction diagnoses among female ACSMs over a 10-year period, from 2014 to 2023. The study’s secondary objective was to identify whether certain socio-demographic or medical variables, including COVID-19 infection history and vaccination status, had an independent association with ovarian dysfunction diagnosis incidence, after adjustment for covariates and potential confounders.

COVID-19 infection history and vaccination status were included in the analysis for several reasons. A sharp increase in the PCOS diagnosis incidence rate was observed from 2020 to 2021, coincident with the onset of the COVID-19 pandemic. Subsequently, several members of Congress expressed concern about a possible relationship between COVID-19 vaccination status and ovarian dysfunction in the U.S. military.^11^ Existing literature suggests that people with existing PCOS may be more susceptible to severe COVID-19 infection, though studies to date do not identify either COVID-19 infection or vaccination as risk factors for ovarian dysfunction conditions.^12,13^

## 2 METHODS

This study used a retrospective cohort study design to examine the incidence of ovarian dysfunction during the surveillance period of January 1, 2014 to December 31, 2023. The study population included all female service members from the active component of the U.S. Armed Forces including the Army, Navy, Marine Corps, Air Force, and Space Force. Individuals serving in the reserves, reservists on active duty, National Guard, and Coast Guard were not included. All data were drawn from the Defense Medical Surveillance System (DMSS), the central repository of medical data for service members. DMSS collects medical encounter data from both the MHS and civilian health care purchased through TRICARE.

Ovarian dysfunction cases were defined using International Classification of Diseases (ICD), 9th and 10th revisions, diagnostic codes. The selected ICD codes included estrogen excess, androgen excess, polycystic ovary syndrome, premature menopause, ovarian failure, and unspecified ovarian dysfunction (**Table 1**). An individual was counted as a case if that person either 1) had at least 1 inpatient encounter with an ovarian dysfunction ICD code in the first or second diagnostic position or 2) had at least 2 outpatient encounters on separate dates with the same ovarian dysfunction code in any diagnostic position.

Socio-demographic and medical covariates examined in relation to ovarian dysfunction included age, race, rank or pay grade, occupation within the military, branch of service, prior COVID-19 infection, COVID-19 vaccination status, and obesity. COVID-19 cases were defined by either a medical encounter with ICD-10-CM code (U07.1) included in any diagnostic position, a positive PCR or antigen test, or a confirmed or probable reportable medical event for COVID-19 infection. COVID-19 vaccination status was defined as having received any dose of the COVID-19 vaccine. To measure both short- and any long-term risks, person-time was divided into 3 categories for COVID-19 infection: 1) never infected, 2) within 180 days after first infection, and 3) more than 180 days after first infection. COVID-19 vaccination status was stratified to the same 3 tiers. Obesity was defined as body mass index (BMI) of 30 or higher with height and weight measurements taken from an annual Periodic Health Assessment (PHA). In addition, individuals were classified as obese if they had a medical encounter with an obesity diagnosis in any diagnostic position.

Person-time was collected from all female ACSMs each year, expressed as person-years (p-yrs). Individuals began contributing person-time on January 1, 2014 or when they entered military service, whichever occurred later. Person-time was censored upon an individual’s first ovarian dysfunction diagnosis. Person-time was also censored upon an individual’s departure from active component service or after December 31, 2023.

Statistical analysis for this study included descriptive statistics and calculation of incidence rates. Incidence rates were expressed as number of cases per 10,000 p-yrs and were calculated for each type of ovarian dysfunction, by year and by covariate. A Poisson regression model was used to identify independent associations of socio-demographic and medical covariates, including COVID-19 infection history and COVID-19 vaccination status, with incident ovarian dysfunction diagnosis, after adjusting for age, race, service branch, rank, occupation, and obesity. For the Poisson regression, the study population was restricted to 2021-2023, as this was the time during which the COVID-19 vaccine was available to service members.

## 3 RESULTS

A gradual increase in ovarian dysfunction incidence was observed from 2014 to 2023, but PCOS was the only ovarian dysfunction condition that increased (Figure). All other conditions did not demonstrate consistent significant change during that period of time. The PCOS incidence rate increased from 32.0 cases per 10,000 p-yrs in 2014 to 62.9 cases per 10,000 p-yrs in 2022. Notably, a sharp incidence increase occurred in the early 2020s, from 39.5 cases per 10,000 p-yrs in 2020 to 54.8 cases per 10,000 p-yrs in 2021, and then to 62.9 cases per 10,000 p-yrs in 2022. The overall PCOS incidence in the active component from 2014 to 2023 was 43.6 cases per 10,000 p-yrs. Because PCOS incidence was responsible for the increase in ovarian dysfunction incidence during the surveillance period, the remainder of this study focuses on PCOS.

From 2014 through 2023, PCOS incidence increased in nearly all demographic subcategories (data not shown). The 25-29-year age group bore the highest incidence burden increase (from 47.3 cases per 10,000 p-yrs in 2014 to 82.8 cases per 10,000 p-yrs in 2023). A steady increase in incidence over 10 years was observed in all race categories, with no significant variation observed between categories. Gradual incidence increases were also observed for all branches of service, with the Air Force and Space Force experiencing the highest incidence burden increase (from 37.1 cases to 74.0 cases per 10,000 p-yrs over 10 years). All enlisted and junior officer pay grades demonstrated a steady increase over 10 years, with no significant variation observed between them. All military occupations also demonstrated a gradual increase, but with health care workers experiencing the highest incidence burden over 10 years (39.1 cases to 74.5 cases per 10,000 p-yrs). **Table 2** summarizes the total PCOS case count and incidence rate from 2014 to 2023 for all socio-demographic groups.

Results from the Poisson regression analysis (**Table 3**) indicate that history of obesity had the strongest association with PCOS, with an adjusted incidence rate ratio (aIRR) of 2.5 and 95% confidence interval (CI) of 2.3-2.6. Age categories of 25-29 years (aIRR 1.9; 95% CI, 1.6-2.3), 20-24 years (aIRR 1.9; 95% CI, 1.6-2.2), and 30-34 years (aIRR 1.3; 95% CI, 1.1-1.6) demonstrated the next highest PCOS associations. Service in the Air Force and Space Force (aIRR 1.3; 95% CI, 1.2-1.4), working in health care (aIRR 1.2; 95% CI, 1.1-1.4), and prior COVID-19 infection (aIRR 1.2; 95% CI, 1.1-1.3) all had modest though significant associations with increased PCOS incidence. No significant differences in PCOS incidence were observed based on race, rank, or COVID-19 vaccination status.

## 4 DISCUSSION

PCOS incidence is not commonly calculated nor tracked annually within the U.S. population, which makes comparisons with the military population difficult. A 2023 retrospective cohort study conducted in the Kaiser Permanente Washington health care system that examined population-level PCOS incidence from 2006 to 2019, however, found an incidence rate of 42.5 cases per 10,000 p-yrs, which was similar to the 43.6 cases per 10,000 p-yrs incidence rate in female ACSMs.^2^ Additionally, the Kaiser study found a gradual upward trend in PCOS incidence among younger patients that was proportionally similar to the upward trend observed in this study, over a similar time frame. PCOS prevalence is a more common metric in literature, but estimates vary greatly, commonly ranging from 7% to 20% of the reproductive age population.^1,2,14^

Multiple explanations for the increase in PCOS diagnoses among female ACSMs from 2014 to 2023 are possible. First, the 2018 International Evidence-based Guideline for the Assessment and Management of Polycystic Ovary Syndrome upheld and refined the 2003 Rotterdam diagnostic criteria for PCOS. Under the 2018 guideline, a PCOS diagnosis could potentially require minimal laboratory testing and no imaging.^15^ A diagnosis could be made if a patient had irregular menstrual cycles or clinical evidence of androgen excess (acne, hirsutism, alopecia), or if other disorders affecting ovulation and hyperandrogenism had been excluded.^16,17^ Irregular cycles are defined as an individual more than 3 years from menarche experiencing cycles less than 21 or more than 35 days apart, or less than 8 cycles per year. The 2018 guideline recommends testing for thyroid-stimulating hormone, prolactin, and follicle-stimulating hormone, at a minimum, to exclude other causes. A patient’s clinical presentation may indicate a need to exclude additional conditions that could present with similar symptoms to PCOS, such as Cushing syndrome, congenital adrenal hyperplasia, or adrenal tumors.^15^ While the 2018 guideline may have resulted in more clinical diagnoses, it does not necessarily explain the relatively sharp increase in PCOS diagnoses starting in 2020, as clinicians are not required to follow it before assigning a PCOS ICD code.

Another, potentially more plausible, explanation is that the COVID-19 pandemic saw an increased use of telehealth encounters during quarantine, less than 2 years after the 2018 guideline was released. This guideline update was more conducive to utilizing virtual health encounters as part of the PCOS diagnostic and management process, given the reduced emphasis on biochemical testing and imaging.^17,18,19^ Future chart review studies could explore whether telehealth and a more clinical diagnostic approach played a role in the increased incidence of PCOS diagnoses among female ACSMs in the early 2020s. Such studies could help assess the clinical decision-making that led to a PCOS diagnosis code assignment for individual patients.

This study found a modest though significant association with COVID-19 infection and increased PCOS incidence (aIRR 1.2; 95% CI, 1.1-1.3). No significant association, however, was found between PCOS incidence and COVID-19 vaccination. Given the risk of severe COVID-19 infection among individuals with PCOS,^12,13^ another hypothesis for the increase in PCOS cases among active component personnel in the early 2020s is that COVID-19 infections may have revealed previously subclinical cases of PCOS by placing those individuals under greater diagnostic scrutiny when they sought medical care.^7,13^

History of obesity had the strongest significant association with increased PCOS incidence after adjustment (aIRR 2.5; 95% CI, 2.3-2.6). Obese individuals are an established high-risk group for PCOS development. It has also been demonstrated that clinicians are significantly more likely to diagnose PCOS in overweight and obese patients, compared to their normal weight and underweight counterparts who may also meet criteria.^20^ Existing PCOS cases are also aggravated by obesity, through worsening insulin resistance and increased androgen production.^1^ Obesity prevalence has gradually risen in both the active duty military and U.S. civilian populations. The proportion of the active duty population that qualifies as obese more than doubled over 10 years, from 10.4% in 2012 to 21.6% in 2022, similar to the time frame of this study.^8^ From 2018 to 2021, obesity prevalence among female active component personnel increased at twice the rate of their male counterparts.^21^ Within this context, increasing PCOS incidence within the military may be more emblematic of the U.S. military’s shifting health burdens, rather than the result of any one extrinsic cause. Factors such as sedentary occupations and lifestyle as well as high caloric diets have been implicated in the etiology of PCOS.^1^ These factors are prevalent in the military and contribute to the increasing burden of obesity and related complications.^8,21^

One of this study’s prominent limitations was that the outcome was operationalized through ICD codes, which may be subject to misclassification bias. For example, the 2023 Kaiser retrospective cohort study determined that 21% of PCOS ICD codes were either assigned without an adequate work-up, or to cases that were not PCOS.^2^ Another hospital-based cohort study conducted over a 12-year period found that PCOS ICD code diagnoses gradually increased each year while the number of patients who potentially qualified for a PCOS diagnosis based on clinical presentation remained steady from year to year.^20^ Our study did not have a parallel chart review component, so knowing whether PCOS diagnosis codes were assigned to female ACSMs in error or without an adequate work-up was beyond its scope. Another limitation was that there are a range of PCOS risk factors this study was unable to examine due to data unavailability, including sedentary lifestyle, high caloric diet, smoking, and family history of PCOS. Finally, the COVID-19 case burden is likely underestimated because data for at-home rapid antigen tests were not available.

Ovarian dysfunction trends among female active component personnel have increased over the past decade, driven almost entirely by increased incidence of PCOS. This study found minimal association between PCOS and COVID-19 infection history and no association between PCOS and COVID-19 vaccination. PCOS incidence was most strongly associated with a history of obesity, which may reflect the changing health burdens in the U.S. military. Additional research is recommended to assess the proportion of PCOS ICD codes that are assigned accurately within the MHS, as this will help further characterize the PCOS burden among the female ACSM population. Further research could then explore the influence of different diagnostic approaches on PCOS incidence, the distribution of PCOS risk factors within the active component population, and the impact of increased PCOS incidence on military readiness.

## Figures and Tables

**Table 1:** Ovarian Dysfunction ICD Codes

ICD-9	ICD-10
256.0. Hyper-estrongenism	E28.0. Estrogen excess
256.1. Ovarian hyperfunction	E28.1. Androgen excess
256.2. Post-ablative ovarian failure	E28.2. Polycystic ovary syndrome
256.31. Premature menopause	E28.310. Symptomatic premature menopause
256.39. Other ovarian failure	E28.319. Asymptomatic premature menopause
256.4. Polycystic ovaries	E28.39. Other primary ovarian failure
256.8. Other ovarian dysfunction	E.28.8. Other ovarian dysfunction
256.9. Unspecified ovarian dysfunction	E28.9. Ovarian dysfunction, unspecified

Abbreviations: ICD, International Classification of Diseases; ICD-9, International Classification of Diseases, 9th Revision; ICD-10, International Classification of Diseases, 10th Revision.

**Figure:**
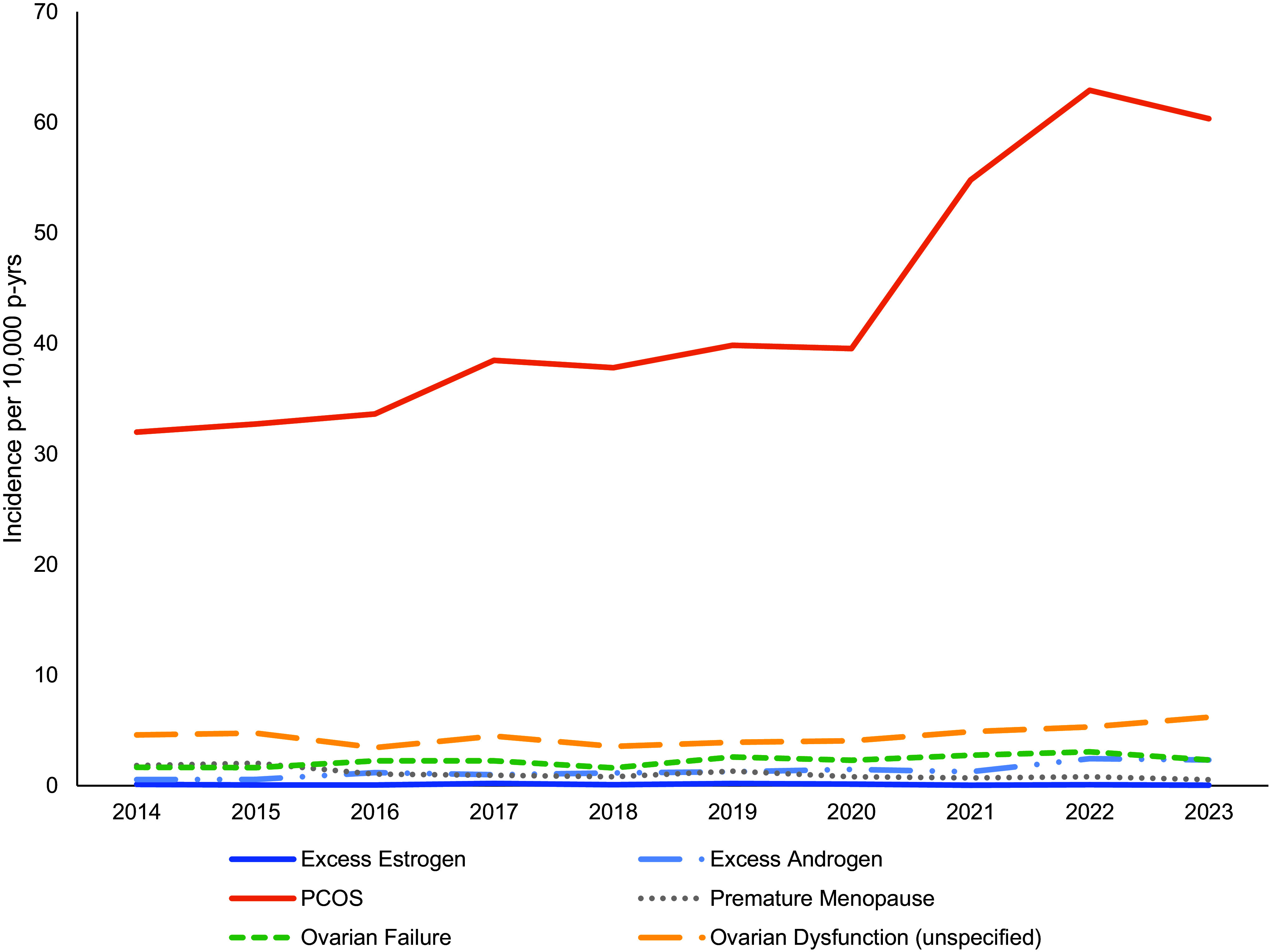
Ovarian Dysfunction Diagnosis Incidence Rates Among Female Active-Component Service Members, U.S. Armed Forces, 2014–2023

**Table 2:** Polycystic Ovarian Syndrome Case Count and Incidence Rates by Demographic Categories, 2014–2023

	Total Case Count	Rate^a^
Total	9,224	43.6
Race and ethnicity		
White, non-Hispanic	3,781	42.3
Black, non-Hispanic	2,425	46.4
Hispanic	1,826	45.8
Other	1,015	39.0
Unknown	177	44.7
Age, y		
<20	334	19.8
20-24	3,521	50.2
25-29	3,120	60.1
30-34	1,499	45.2
35-39	607	27.4
40-44	117	11.0
45-49	22	5.0
50+	4	1.8
Service branch		
Army	2,784	40.0
Navy	2,705	42.9
Air Force/Space Force	3,338	52.6
Marine Corps	397	25.7
Rank		
Junior enlisted (E1-E4)	4,462	46.9
Senior enlisted (E5-E9)	3,390	45.2
Junior officer (O1-O3)	1,149	43.0
Senior officer (O4-O10)	196	15.3
Warrant officer (W)	27	16.1
Occupation		
Combat-specific	212	38.2
Armor/motor transport	233	35.1
Pilot/air crew	114	34.8
Repair/engineering	1,832	43.4
Communications/intelligence	3,035	45.1
Health care	2,066	52.6
Other	1,732	36.8

Abbreviations: y, years; p-yrs, person-years.

^a^Rate per 10,000 p-yrs.

Note: The most significant PCOS diagnosis incidence increases from 2014-2023 were observed among ages 20-39 years, Air Force/Space Force, enlisted and junior officer pay grades, and health care occupations. All race and ethnicity subcategories experienced increased incidence with no significant variation.

**Table 3:** Comparison of Ovarian Dysfunction Diagnosis Incidence by Socio-Demographic and Medical Factors

	aIRR	95% LL	95% UL	*P*-value
Race and ethnicity				
White, non-Hispanic	Reference	-	-	-
Black, non-Hispanic	1.0	0.9	1.1	0.8501
Hispanic	1.0	0.9	1.1	0.7782
Other	0.9	0.8	1.0	0.0342
Unknown	1.1	0.8	1.4	0.5893
Age, y				
<20	Reference	-	-	-
20-24	1.9	1.6	2.2	<0.0001
25-29	1.9	1.6	2.3	<0.0001
30-34	1.3	1.1	1.6	0.0041
35-39	0.8	0.6	1.0	0.0197
40+	0.3	0.2	0.4	<0.0001
Service branch				
Army	Reference	-	-	-
Navy	1.0	1.0	1.1	0.3740
Air Force/Space Force	1.3	1.2	1.4	<0.0001
Marine Corps	0.8	0.6	0.9	0.0007
Rank				
Enlisted	Reference	-	-	-
Officer	0.9	0.9	1.0	0.2148
Occupation				
Combat-specific	0.8	0.7	1.0	0.0866
Armor/motor transport	0.8	0.6	1.0	0.0187
Pilot/air crew	0.8	0.6	1.1	0.1367
Repair/engineering	0.9	0.8	1.0	0.0107
Communications/intelligence	Reference	-	-	-
Health care	1.2	1.1	1.4	<0.0001
Other	0.8	0.8	0.9	<0.0001
Prior diagnosis of COVID-19 infection				
Yes	1.2	1.1	1.3	<0.0001
No	Reference	-	-	-
Prior COVID-19 vaccination				
Yes, within past 180 days	0.9	0.8	1.0	0.1578
Yes, not within past 180 days	1.0	1.0	1.1	0.3886
No, never	Reference	-	-	-
Prior obesity diagnosis, BMI				
Yes	2.5	2.3	2.6	<0.0001
No	Reference	-	-	-

Abbreviations: aIRR, adjusted incidence rate ratio; LL, lower limit; UL, upper limit; y, years; COVID-19, coronavirus disease 2019; BMI, body mass index.

Note: Results of the Poisson regression model expressed as adjusted incident rate ratios with 95% confidence intervals. The model adjusted for race and ethnicity, age, service branch, rank or pay grade, occupation within the military, prior COVID-19 diagnosis, COVID-19 vaccination status, and history of obesity.

## AUTHOR AFFILIATIONS

Uniformed Services University of the Health Sciences, Bethesda, MD: LT Douthitt; Epidemiology and Analysis Branch, Armed Forces Health Surveillance Division, Defense Health Agency, Silver Spring, MD: Ms. Murray, Dr. Stahlman
